# Contactin-1 Is Required for Peripheral Innervation and Immune Homeostasis Within the Intestinal Mucosa

**DOI:** 10.3389/fimmu.2020.01268

**Published:** 2020-06-26

**Authors:** Marisol Veny, Daniela Grases, Karolina Kucharova, Wai Wai Lin, Jennifer Nguyen, Sarah Huang, Carl F. Ware, Barbara Ranscht, John R. Šedý

**Affiliations:** ^1^Infectious and Inflammatory Disease Center, Sanford Burnham Prebys Medical Discovery Institute, La Jolla, CA, United States; ^2^Neuroscience and Aging Research Center, Sanford Burnham Prebys Medical Discovery Institute, La Jolla, CA, United States

**Keywords:** contactin-1, hypothalamus pituitary adrenal (HPA) axis, mucosal immunity, neuro-immune crosstalk, immune homeostasis, T cell, small intestine, villi

## Abstract

Neuronal regulation of diverse physiological functions requires complex molecular interactions in innervated tissues to maintain proper organ function. Here we show that loss of the neuronal cell surface adhesion/recognition molecule Contactin-1 (*Cntn1)* directly impairs intestinal function causing wasting that subsequently results in global immune defects. Loss of *Cntn1* results in hematologic alterations and changes in blood metabolites associated with malnourishment. We found thymus and spleen of *Cntn1*-deficient animals atrophied with severe reductions in lymphocyte populations. Elevated thymic *Gilz* expression indicated ongoing glucocorticoid signaling in *Cntn1*-deficient animals, consistent with the malnourishment phenotype. Intestinal Contactin-1 was localized to neurons in the villi and the submucosal/myenteric plexus that innervates smooth muscle. Loss of *Cntn1* was associated with reduced intestinal *Bdnf* and *Adrb2*, indicating reduced neuromuscular crosstalk. Additionally, loss of *Cntn1* resulted in reduced recruitment of CD3^+^ T cells to villi within the small intestine. Together, these data illustrate the critical role of Contactin-1 function within the gut, and how this is required for normal systemic immune functions.

## Introduction

Contactin-1 (CNTN1) is the prototype of neural cell adhesion/recognition molecules with an extracellular domain consisting of six aminoterminal immunoglobulin-like and four fibronectin-like structural domains that are attached to the plasma membrane through a glycosylphophatidylinsitol (GPI) moiety (1, 2). Since its discovery critical roles for Contactin-1 have been shown in the formation of neuronal microcircuitries and in myelin where it orchestrates the organization of myelin membrane domains around nodes of Ranvier and confers signaling between axons and oligodendrocytes during myelin formation (3–8). Specifically, Contactin-1 expressed at paranodes is required to form axoglial attachment sites next to the nodes of Ranvier that serve to segregate voltage-gated sodium channels from the potassium channels underneath the myelin. Loss of paranodal attachment in mice with inactivated *Cntn1* gene expression compromises saltatory nerve conduction and rapid propagation of action potentials (7, 9, 10). Seropositivity for Contactin-1 antibodies in humans is diagnostic for forms of Chronic inflammatory demyelinating polyneuropathy (CIDP) and associated with nodopathy subtypes (11). Humans harboring homozygous *CNTN1* mutations develop congenital myopathy resulting in lethality within 1 month of birth (10). These *CNTN1* mutations were predicted to lead to loss of function through generation of a truncated protein that is degraded by non-sense-mediated decay. Additionally, upregulated levels of *CNTN1* are associated with several types of cancers (12, 13).

In the mouse model of systemic *Cntn1*-deficiency, animals develop normally until approximately P10, then fail to thrive with an ataxic and anorexic phenotype and succumb predictably at P16–P18 (6, 14). Since in healthy animals Contactin-1 expression is restricted to neural tissues and neurons innervating peripheral organs, we hypothesized that the failure to thrive phenotype relates to disturbances in neuron – target communication that in turn affect organ function. Here we address for the first time the role of Contactin-1 in peripheral organs and report the hematologic phenotype of *Cntn1*-deficient animals. Our data establish abnormalities in blood metabolites as well as hematologic imbalances in the blood, thymus, and spleen. Additionally, we show elevated thymic expression of glucocorticoid-induced leucine zipper (Gilz; *Tsc22d3*), consistent with enhanced glucocorticoid signaling and a resultant thymic involution phenotype. However, anti-inflammatory interventions did not rescue the failure to thrive phenotype of *Cntn1*-deficient animals, rendering contributions of immune dysfunctions unlikely. While the failure to thrive phenotype of *Cntn1*-deficient animals is consistent with intestinal malabsorption, diverse dietary interventions did not improve health or extend life span. Remarkably, however, we found reduced innervation of intestinal villi and the submucosal/myenteric plexus along with reduced numbers of CD3^+^ T cells within the intestinal mucosa. These results establish a gut-intrinsic requirement for Contactin-1 in intestinal innervation that is essential for appropriate food uptake and in turn affects immune homeostasis and organ function.

## Materials and Methods

### Mice

All animal husbandry, specimen collection, and *in vivo* treatments were approved by the Sanford Burnham Prebys Medical Discovery Institute Animal Care and Use Committee (IACUC). The derivation of *Cntn1*^−/−^ animals was described previously (6). In this study wild-type and homozygous *Cntn1*-deficient mice were analyzed and compared between postnatal day P0 and P18. PCR analysis was conducted to confirm the genotype of the mice using the primers Cntn Forward (AGTGTCTGAGGAGGACAAAGGATTTGG), Cntn Reverse (GTGGGTGGAGAGCATTACTTGTAAACTGG), and Cntn Neo (GCCTTCTATCGCCTTCTTGACGAGTTC). PCR reactions included a hot start of 5 min 95°C and 30 cycles of 1 min 95°C/1 min 67°C/2 min 72°C, and a final step of 10 min 72°C, followed by 4°C reaction stop.

### Blood Analysis

Blood was collected by cardiac puncture from P16 animals. A profile of white blood cells (WBC), red blood cells (RBC), and platelets was obtained using VetScan HM II Hematology analyzer using 100 μl of blood. WBC: Count with 3-part differential (lymphocytes, monocytes, granulocytes). RBC: Red Blood Cell count and indices. RDW: Red Cell Distribution Width. Measurements of clinical chemical and immunological parameters as well as of electrolytes were obtained using VetScan VS2 analyzer analyzer using 100 μl of blood.

### Histochemistry and TUNEL Staining

Whole spleens, thymuses, livers, and colons were fixed in formalin fixative and embedded in paraffin. Serial sections were stained with hematoxylin and eosin to analyze tissue architecture or apoptotic events were visualized for TUNEL reactivity using the ApopTag Peroxidase *In Situ* Apoptosis Detection Kit according to the manufacturer's instructions (Millipore, Temecula, CA). Whole mount histology sections were scanned using ScanScope® AT2, imaged with Aperio eSlide Manager (Leica Biosystems, Buffalo Grove, IL). TUNEL^+^ events were quantified using the Nuclear v9 algorithm within the Aperio AT2 scanner.

### Flow Cytometry

Whole spleens and thymuses were disaggregated into single cell suspensions and stained for surface receptors for 30 min, followed by washing twice with buffer and flow cytometric analysis. Whole intestines were flushed twice with Hank's buffered saline solution (HBSS) and washed twice in HBSS to remove debris. Intestines were then digested with 1 mg/ml collagenase IV and 40 μg/ml DNAse I shaking for 15 min 37°C to prepare single cell suspensions, followed by washing in HBSS. Data was collected using a LSRFortessa X20 (BD Biosciences, San Jose, CA). Anti-γδTCR FITC, anti-CD44 PerCP-Cy5.5, anti-CD62L APC, anti-CD19 Alexa 700, anti-TCRβ APC-Cy7, anti-CD4 PE-610, anti-CD69 eFluor450, anti-F4/80 APC, anti-MHCII Alexa 700, and anti-CD11b PE-Cy7 were from Thermo Fisher Scientific (Carlsbad, CA). Anti-CD8 BV 605, anti-CD25 PE, and anti-Ly6G BV 421 were from Biolegend (San Diego, CA). Anti-NK1.1 FITC and anti-CD11c PE were from BD Biosciences (San Jose, CA).

### Gene Expression Analysis

Whole small intestine, spleen, and thymic stromal cells were used to prepare RNA. Whole small intestine and spleen tissue was homogenized in Qiagen RLT buffer using the Bullet Blender cell disruptor according to manufacturer's tissue specific instructions (Zymo Research, Irvine, CA). Disaggregated thymuses were digested with 1 mg/ml collagenase IV and 40 μg/ml DNAse I shaking for 30 min 37°C, followed by separation of soluble non-stromal cell fraction from undigestible stromal cell fraction. Total RNA was prepared using the Qiagen RNeasy Mini kit (Qiagen, Valencia, CA) and RNA was reverse transcribed into cDNA using the iScript^TM^ cDNA synthesis kit (Bio-Rad Laboratories, Hercules, CA) according to the manufacturer's instructions. Expression of specific transcripts was measured using SYBR Green Master Mix (Bio-Rad Laboratories, Hercules, CA). Reactions were carried out in clear 384 plates using an ABI® 7900HT Real-Time PCR System (Thermo Fisher Scientific, Carlsbad, CA). Relative expression was determined compared to *L32* and calculated as follows: 2^−Δ*Ct*^, where Ct = cycle number, ΔCt = Ct(target gene) – Ct(*L32*). The following primer pairs were used: *Foxn1* (ATGGTGTCGCTACTCCCTCC, AGGCACAAACGACGAGCAG), *Ccl19* (TGTGGCCTGCCTCAGATTAT, AGTCTTCCGCATCATTAGCAC), *Ccl21* (TCCAAGGGCTGCAAGAGA, TGAAGTTCGTGGGGGATCT), *Il7* (CGCAGACCATGTTCCATGT, TCTTTAATGTGGCACTCAGATGAT), *Tsc22d3* (AACACCGAAATGTATCAGACCC, GTTTAACGGAAACCAAATCCCCT), *Lta* (CCACCTCTTGAGGGTGCTTG, CATGTCGGAGAAAGGCACGAT), *Tnf* (CCCTCACACTCAGATCATCTTCT, GCTACGACGTGGGCTACAG), *Il1b* (TGTAATGAAAGACGGCACACC, TCTTCTTTGGGTATTGCTTGG), *Ifng* (ATGAACGCTACACACTGCATC, CCATCCTTTTGCCAGTTCCTC), *Csf2* (GGCCTTGGAAGCATGTAGAGG, GGAGAACTCGTTAGAGACGACTT), *Vcam1* (AGTTGGGGATTCGGTTGTTCT, CCCCTCATTCCTTACCACCC), *Icam1* (GGCATTGTTCTCTAATGTCTCCG, TGTCGAGCTTTGGGATGGTAG), *Ccl2* (GAGCATCCACGTGTTGGCT, TGGTGAATGAGTAGCAGCAGGT), *Ccl3* (TTCTCTGTACCATGACACTCTGC, CGTGGAATCTTCCGGCTGTAG), *Cntn1* (TTGTCTAGGAGACTTTACCTGGC, AAATGGTATTGATTGGCTGCTCT), *Bdnf* (TCATACTTCGGTTGCATGAAGG, AGACCTCTCGAACCTGCCC), *Adrb2* (CGAGCTGAGTGTGCAGGAC, GACTCCTGGAAGCTTCATTCA).

### Immunofluorescence Histology

Spleens, thymuses and Intestines were harvested fresh, directly embedded and frozen in OCT compound (Sakura, Torrance, CA), and 10 μm sections were cut at −20°C (Leica CM 3050 S). Frozen sections were left at RT to air dry for 5 min, fixed with cold acetone for 10 min (spleen) or incubated with 0.1 M PBS followed by 15 min postfixation in 4% paraformaldehyde in 0.1 M PBS (intestine), and subsequently washed 3 times with PBS for 10 min. Sections were incubated in blocking buffer (5% normal donkey serum and 0.8% Triton X-100) for 1 h at RT. Primary antibodies were diluted in blocking buffer and incubated overnight at 4°C, washed 3 times with PBS for 10 min and then incubated with secondary antibodies diluted in blocking buffer as needed for 1 h at RT. Slides were washed 3 times with PBS 10 min each, air-dried and cover-slipped with ProLong Gold (Thermo Fisher Scientific, Carlsbad, CA), and imaged on a confocal microscope (Zeiss LSM-710). Anti-CD3 Alexa 488, Phalloidin Green 488, and anti-Podoplanin Alexa 488 were from Biolegend (San Diego, CA). Anti-B220 eFluor570 and donkey anti-goat Alexa 568 were from Thermo Fisher Scientific (Carlsbad, CA). Anti-pan neurofilament, anti-NF200, and anti-Contactin were from Neuromics (Edina, MN). Donkey anti-mouse Alexa 647 was from Jackson Immunoresearch (West Grove, PA).

### *In vivo* Treatments

Animals were dosed with intraperitoneal injections of the indicated treatments at P10 and P12 except as noted and monitored daily for improvement of the wasting phenotype. Anti-TNF (100 μg/animal) was from Thermo Fisher Scientific (Carlsbad, CA). Anti-CD4 (100 μg/animal) was from Bio X Cell (West Lebanon, NH). TNFR Fc (75 μg/animal) was produced as previously described (15). Clodronate liposome solution (50 μl/animal) was from Encapsula Nanosciences (Brentwood, TN). Dexamethasone was injected daily from P9 (100 μg/animal) was a gift from Jesus Rivera-Nieves. GTS-21 (40 μg/animal) was from Sigma-Aldrich (Saint Louis, MO).

## Results

### Hematologic Alterations in *Cntn1*-Deficient Mice Are Consistent With Malnourishment

**Figure 1:**
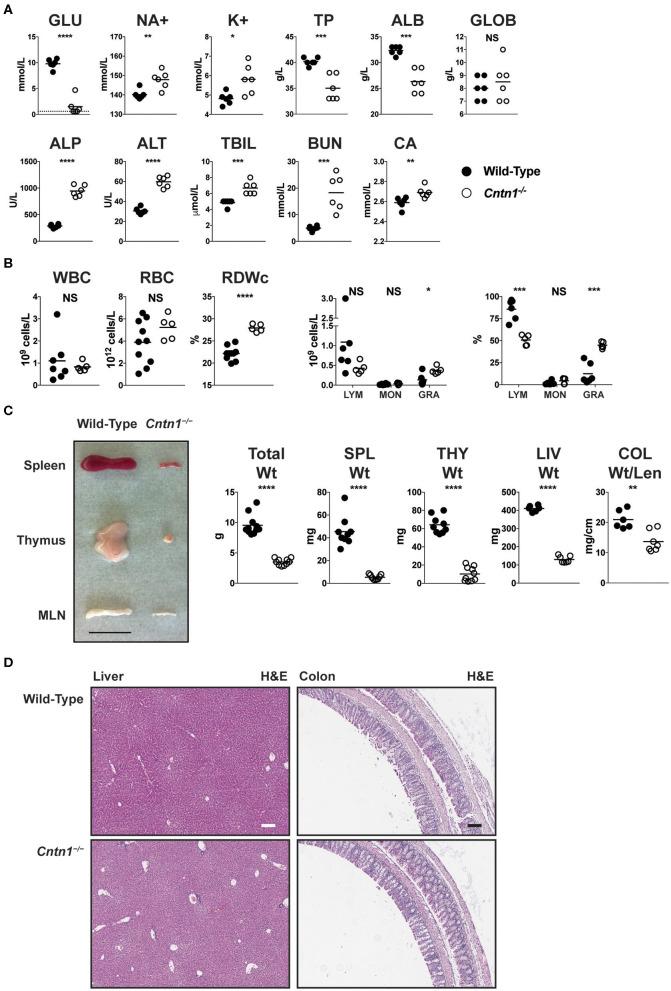
*Cntn1*^−/−^ animals show hematologic and lymphoid abnormalities prior to expiration. **(A)** Blood from P16 wild-type and *Cntn1*^−/−^ animals was analyzed for the indicated chemical paramaters (GLU, glucose; NA+, sodium; K+, potassium; TP, total protein; ALB, albumin; GLOB, globulin; ALP, alkaline phosphatase; ALT, alanine aminotransferase; TBIL, bilirubin; BUN, urea; CA, Calcium). **(B)** Blood from P16 wild-type and *Cntn1*^−/−^ animals was analyzed for the indicated cellular paramaters (WBC, white blood cells; RBC, red blood cells; RDWc, RBC distribution width; LYM, lymphocytes; MON, monocytes; GRA, granulocytes). **(C)** Representative images of spleen, thymus and mesenteric lymph nodes (MLN) harvested from P17 wild-type and *Cntn1*^−/−^ animals are shown. Graphs show weight of animals, spleens, thymuses, livers, and colon weight/length. **(D)** Representative images of livers (left) and colons (right) from wild-type (top) and *Cntn1*^−/−^ (bottom) at 10 × (H&E). White and black scale bars represent 100 μm. Analysis of data by Student's *t*-test. NS, not significant; *p < 0.05; **p < 0.01, ***p < 0.001; ****p < 0.0001.

Early lethality in *Cntn1*-deficient animals precluded efforts to study the function of Contactin-1 in adult mice. Since wasting of *Cntn1*-deficient animals correlated with the time of weaning, we attempted to administer nutrition to *Cntn1*^−/−^ *pups* through assisted manual feeding or parenteral administration (intraperitoneal) of nutrients. However, these efforts all proved unsuccessful and did not improve the wasting phenotype or survival of *Cntn1*^−/−^ animals past 3 weeks of age. We next examined blood chemistry and hematologic signatures to identify parameters that could indicate the basis for weight loss in *Cntn1*^−/−^ animals. *Cntn1*-deficient mice were significantly hypoglycemic, hypernatremic, and hyperkalemic, consistent with undernourishment and dehydration (Figure 1A). In the blood of *Cntn1*-deficient animals we observed elevated liver enzymes, bilirubin, urea, and anisocytosis consistent with nutrient deprivation and liver disease. Furthermore, measurements of white blood cells in *Cntn1*-deficient indicated lymphopenia and granulocytosis (Figure 1B). Gross analysis of lymphoid tissues in *Cntn1*-deficient animals prior to expiry (P16) revealed visually atrophied thymus and spleen compared to wild-type animals (Figure 1C). In *Cntn1*-deficient mice the livers and colons were also reduced in size compared to wild-type animals, though no gross histological changes in these organs were noted (Figure 1D). The hematologic changes in *Cntn1*^−/−^ animals indicated that wasting was likely due to malnutrition. This raised the puzzling question why these animals are unable to respond to dietary supplementation and how lymphoid tissue defects relate to the wasting phenotype.

### Thymic Involution in *Cntn1*-Deficient Mice Correlates With Glucocorticoid Signaling

**Figure 2:**
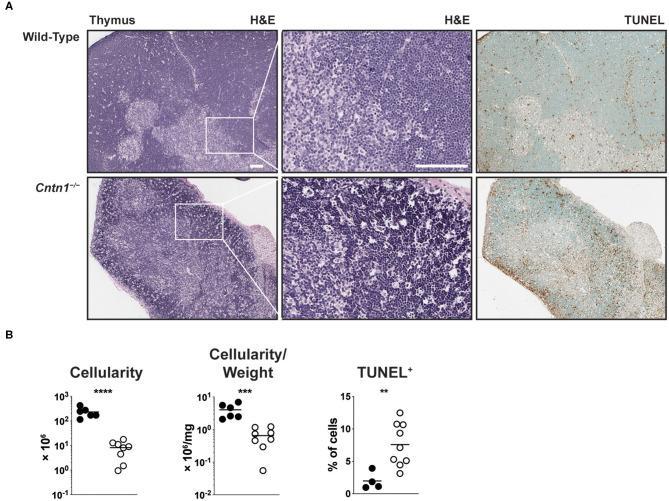
*Cntn1*^−/−^ animals show thymic atrophy prior to expiration. **(A)** Representative images of P16 thymuses from wild-type (top) and *Cntn1*^−/−^ (bottom) at 4 × (H&E, upper panels; TUNEL, lower panels) or 10 × (H&E, middle panels). White scale bars represent 100 μm. **(B)** Graphs show thymus cellularity (left), cellularity normalized to organ weight (middle), or TUNEL^+^ cells (right) from individual wild-type and *Cntn1*^−/−^ animals. Analysis of aggregated data by Student's *t*-test. **p < 0.01, ***p < 0.001; ****p < 0.0001.

**Figure 3:**
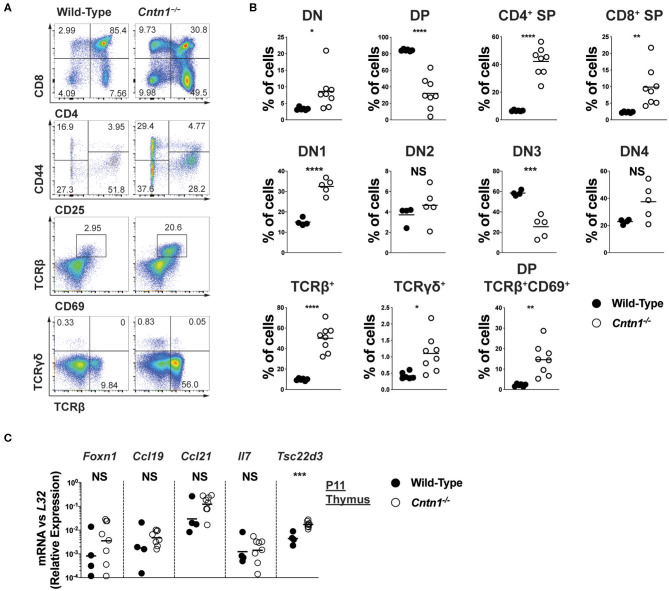
Altered thymocyte development in *Cntn1*^−/−^ mice is associated with elevated glucocorticoid signaling **(A,B)**. Representative FACS analysis of wild-type (left panels) and *Cntn1*^−/−^ (right panels) thymuses. Total live thymocytes analyzed for the frequency of DN, DP, and CD4^+^ and CD8^+^ SP cells (first row), CD4^−^CD8^−^ thymocytes analyzed for the frequency of DN subsets using CD25 vs CD44 (second row), CD69 vs. TCRβ in total thymocytes (third row), and TCRβ vs. TCRγδ in total thymocytes (fourth row) **(A)**. The frequency of thymocyte populations from individual wild-type and *Cntn1*^−/−^ animals are graphed in **(B)**. Analysis of aggregated data by Student's *t*-test **(C)**. P11 enriched thymic stromal cells from wild-type and *Cntn1*^−/−^ animals were harvested for RNA and analyzed for the indicated genes by QRT-PCR. Graphs show transcript abundance relative to *L32*. Individual transcripts were compared by Student's *t*-test. NS, not significant; *p < 0.05; **p < 0.01, ***p < 0.001; ****p < 0.0001.

Atrophied thymuses of P16 *Cntn1*-deficient animals showed a greatly reduced cortical area compared to wild-type animals and a “starry sky” pattern previously ascribed to thymic macrophages that have phagocytosed cellular fragments (Figure 2A) (16). We confirmed an increased frequency of apoptotic-like events in *Cntn1*^−/−^ thymuses compared to wild-type using TUNEL staining (Figures 2A,B). These histologic changes correlated with altered frequencies of thymocyte populations, including greatly reduced CD4^+^CD8^+^ double positive (DP) cells, and overall reduced total numbers of DP and CD4^+^ single positive thymocytes in *Cntn1*-deficient animals compared to wild-type (Figures 3A,B). While the frequency of CD4^−^CD8^−^ double negative (DN) thymocytes was not significantly different in *Cntn1*-deficient animals, altered frequencies of DN subpopulations indicated inefficient thymocyte maturation (Figures 3A,B). To identify underlying causes to thymocyte defects in *Cntn1*-deficient animals, we analyzed thymic stroma transcripts from P11 mice, before changes in weight were observed. Transcripts encoding the transcription factor *Foxn1* required for differentiation of thymic epithelial cells, and for the thymocyte supportive cytokines *Ccl19, Ccl21*, and *Il7* were not significantly different between wild-type and *Cntn1*-deficient animals (Figure 3C) (17). The thymus is particularly sensitive to systemic stresses including malnutrition, which results in glucocorticoid release that in part facilitates liver and muscle gluconeogenesis (18, 19). Glucocorticoid signaling in the thymus induces thymocyte apoptosis and overall reduction of thymocyte frequencies (20). We thus examined levels of mRNA encoding the leucine zipper-containing protein Glucocorticoid-Induced Leucine Zipper (Gilz; *Tsc22d3*) that homo- and hetero-dimerizes with transcription factors including NF-κB, AP-1, and C/EBP, and is induced following glucocorticoid receptor activation (21–23). In *Cntn1*-deficient thymuses, In contrast to *Ccl19, Ccl21, Il7*, and *Foxn1, Tsc22d3* levels were significantly elevated in *Cntn1*-deficient thymuses, indicating ongoing glucocorticoid signaling. Together, these data are consistent with the suggestion that thymic defects observed in *Cntn1*-deficient animals may represent a response to hypoglycemia-induced glucocorticoid signaling, although we cannot exclude the alternative possibility that Cntn1 directly regulates glucocorticoid signaling in the thymus.

### Wasting Phenotype in *Cntn1^−/−^* Animals Is Unaffected by Immune-Ablating Therapies

**Figure 4:**
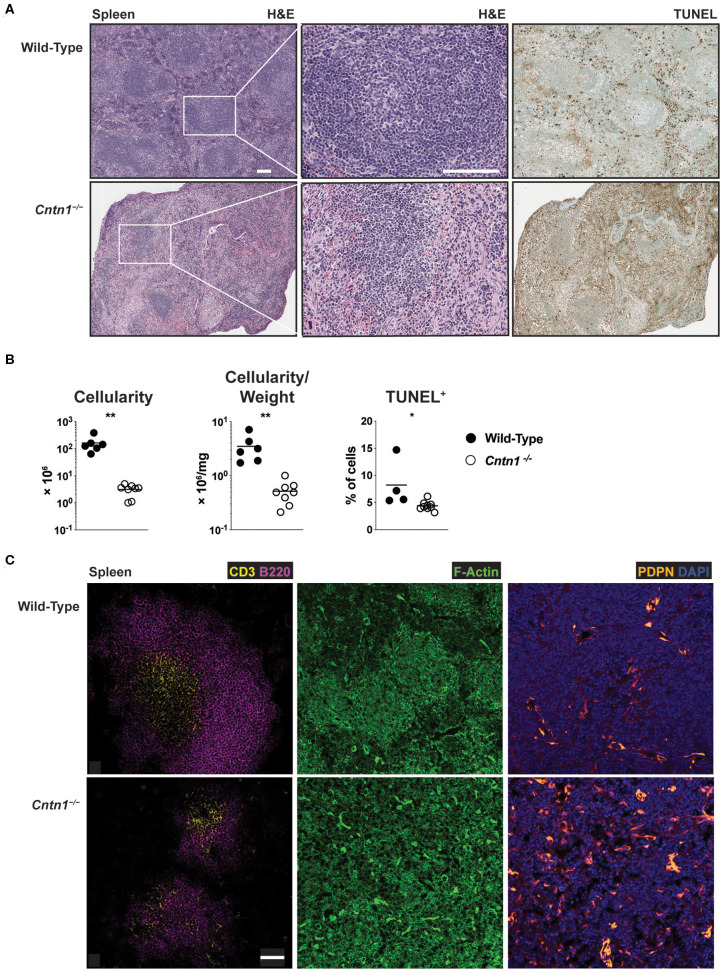
Disorganized architecture of *Cntn1*-deficient spleens **(A)**. Representative images of spleens from wild-type (left) and *Cntn1*^−/−^ (right) at 4 × (H&E, upper panels; TUNEL, lower panels) or 10 × (H&E, middle panels). White scale bars represent 100 μm **(B)**. Graphs show spleen cellularity (left), cellularity normalized to organ weight (middle), or TUNEL^+^ cells (right) from individual wild-type and *Cntn1*^−/−^ animals. Analysis of aggregated data by Student's *t*-test **(C)**. Representative immunofluorescence images of spleens from wild-type (top) and *Cntn1*^−/−^ (bottom) costaining for CD3 (yellow) and B220 (magenta) (left panels, 4 ×), F-actin (phalloidin-green) (middle panels, 4 ×), and PDPN (orange) and DAPI (blue) (right panels, 4 ×). White scale bar represents 50 μm. *p < 0.05; **p < 0.01.

**Figure 5:**
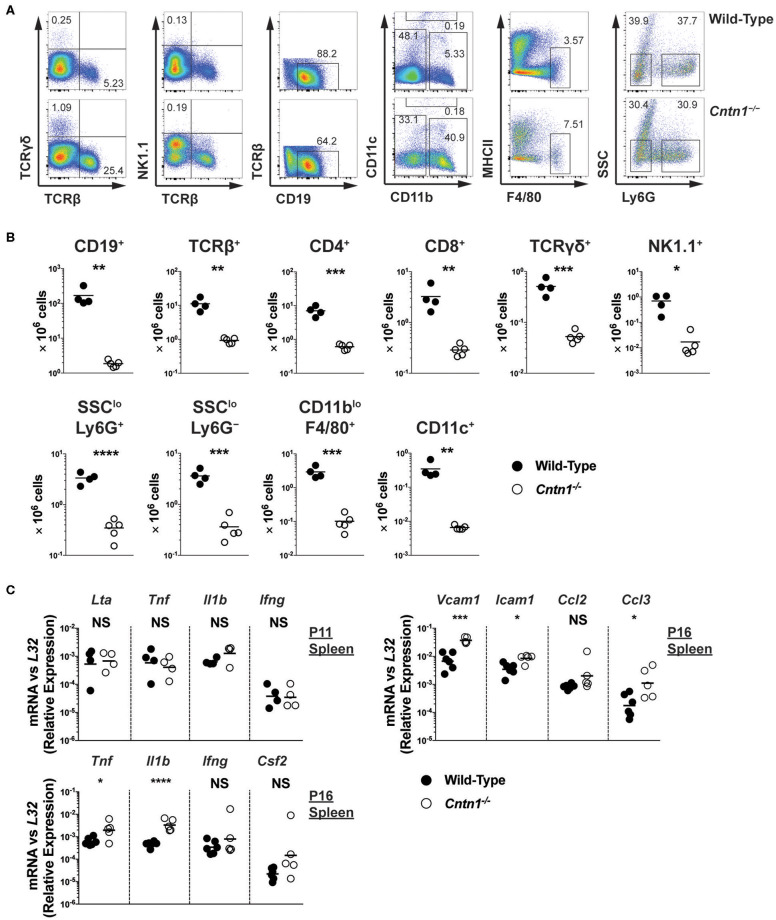
Hypocellularity and increased inflammatory signaling in *Cntn1*^−/−^ spleens **(A,B)**. Representative FACS analysis of wild-type (top) and *Cntn1*^−/−^ (bottom) P16 spleens. Total live splenocytes analyzed for the frequency of αβ T cells (TCRβ^+^) and γδ T cells (TCRγδ^+^), natural killer cells (TCRβ^−^NK1.1^+^), TCRβ^−^TCRγδ^−^ cells for the frequency of B cells (CD19^+^), TCRβ^−^NK1.1^−^ cells for the frequency of dendritic cells (CD11c^hi^), TCRβ^−^NK1.1^−^CD11c^−^CD11b^lo^ cells for the frequency of macrophages (F4/80^+^MHCII^lo^), and TCRβ^−^NK1.1^−^CD11c^−^CD11b^hi^ cells for the frequency of neutrophils (Ly6G^+^SSC^lo^) and monocyte containing populations (Ly6G^−^SSC^lo^) **(A)**. Lineage gating was structured according to previously reported analysis (24). The frequency of individual splenocyte populations are graphed in **(B)**. Analysis of data by Student's *t*-test **(C)**. P11 and P16 splenic tissue from wild-type and *Cntn1*^−/−^ animals was harvested for RNA and analyzed for the indicated genes by QRT-PCR. Graphs show transcript abundance relative to *L32*. Individual transcripts were compared by Student's *t*-test. NS, not significant; *p < 0.05; **p < 0.01; ***p < 0.001; ****p < 0.0001.

Spleens from P16 *Cntn1*-deficient animals were disorganized compared to wild-type animals, with diminished lymphocyte-containing white pulp areas and reduction in TUNEL staining in these areas (Figures 4A,B). T and B cells were less numerous and poorly segregated in *Cntn1*^−/−^ spleens compared to wild type (Figure 4C). Additionally, actin and podoplanin staining was more prevalent in *Cntn1*^−/−^ spleens due to the absence of lymphocytes (Figure 4C). In addition to the changes in the frequencies of diverse hematopoietic cell populations within the spleen in *Cntn1*^−/−^ mice (Figure 5A), the overall numbers of all hematopoietic splenocytes were significantly reduced in *Cntn1*^−/−^ this tissue (Figures 4B, 5B). We examined the levels of inflammatory cytokines in the spleen to determine whether immune signaling may induce these cellular changes. In P11 animals prior to overt weight loss defects, no significant differences were observed between wild-type and *Cntn1*^−/−^ spleens (Figure 5C). However, at P16, transcripts encoding inflammatory cytokines Tumor Necrosis factor-α (TNF-α) and Interleukin-1β (IL-1β) (*Il1b* and *Tnf* ) and their downstream targets Vascular Cell Adhesion Molecule 1, Intracellular Adhesion Molecule 1, and the cytokine Macrophage Inflammatory Protein 1α (*Vcam1, Icam1*, and *Ccl3*) were increased within the spleens of *Cntn1*-deficient animals. The reduction in splenocyte numbers in *Cntn1*^−/−^ animals is likely a result of the hypoglycemic response similar to thymic involution (20). However, it remains possible that elevated TNF-α and IL-1β contributes to inflammation-induced wasting.

We next sought to prevent post-natal lethality in *Cntn1*-deficient animals through selective blockade or depletion of lymphocyte populations or effector proteins that may mediate inflammation-induced cachexia (Table 1). We first sought to deplete excess TNF-α using antibody blockade or using p60 TNFR1-Fc proteins. Additionally, since T cells may be a major source of TNF-α, we used CD4^+^ cell antibody depletion to prevent inflammatory cytokine secretion. Finally, we depleted macrophages using clodronate liposomes to prevent inflammatory activation of lymphocytes. However, neither or these approaches altered lethality. We next used dexamethasone to globally suppress immune responses and early lethality. Again, steroid treatment did not alter progression to lethality. Finally, we reasoned that Contactin-1 may be required for optimal vagus nerve stimulation, and that in *Cntn1*^−/−^ animals unrestricted hypothalamic nervous stimulation could promote inflammatory signaling and early lethality. However, our attempts to reduce inflammation by activating α7 nicotinic acetylcholine receptors with the agonist GTS-21 again did not alter lethality (25). Thus, we conclude that despite apparent increases in inflammatory markers in the spleen, these do not induce a systemic cachectic response, and thus are unlikely to cause the wasting phenotype.

**Table 1:** Immune-ablating therapies fail to prolong survival in *Cntn1*-deficiency.

**Treatment**	**Survival/normo-weight at day 17**	
	***Cntn1^**+/+**^***	***Cntn1^**−/−**^***
Anti-TNF: (blocks TNFα)	3/3	0/2
TNFR Fc: (blocks TNFα)	4/4	0/4
Clodronate liposomes: (depletes F4/80^+^ macrophages)	5/5	0/7
Anti-CD4: (depletes CD4^+^ T cells)	4/4	0/4
Dexamethasone: (systemic immunosuppressant)	5/5	0/4
GTS-21 cholinergic agonist: (activates α7 nAChR)	5/5	0/4

### Cntn1 Is Required for Nervous and Immune Homeostasis Within the Intestine

**Figure 6:**
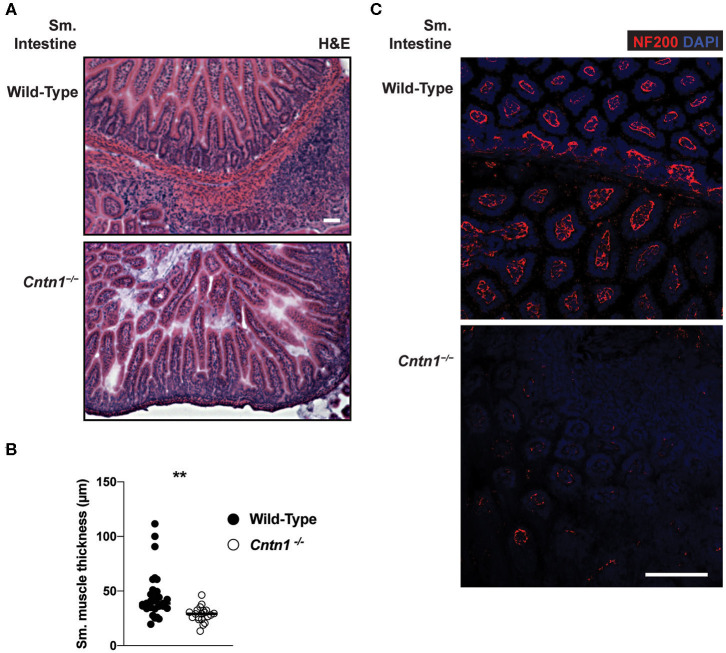
Altered neuromuscular homeostasis in *Cntn1-/-* small intestine **(A)** Representative images of P11 small intestine from wild-type (top) and *Cntn1*^−/−^ (bottom) at 10 × (H&E). White scale bars represent 50 μm **(B)**. Graph shows measurements of smooth muscle thickness in histology sections. From wild-type and *Cntn1*^−/−^ animals. Analysis of aggregated data by Student's *t*-test **(C)** Representative immunofluorescence images of small intestine from wild-type (top) and *Cntn1*^−/−^ (bottom) costained for NF200 (red) and DAPI (blue) White scale bar represents 50 μm. **p < 0.01.

Since defects in lymphoid cells were not ikely to be the primary cause for wasting and consequent lymphoid tissue atrophy in *Cntn1*^−/−^ animals, we next examined the possibility that the wasting phenotype is due to intestinal malabsorption. Gross examination of the upper digestive tract of P14 *Cntn1*^−/−^ animals showed a lack of visible milk content and reduced size of the small bowel compared to wild-type animals, similar to the colon (data not shown). Histological examination of the small intestine of *Cntn1*^−/−^ animals revealed slighty atrophied villi compared to wild-type animals with no obvious signs of inflammation (Figure 6A). Additionally, lymphoid structures were ill-defined in *Cntn1*^−/−^ tissues compared to those in wild-type intestines. In *Cntn1*^−/−^ animals villus atrophy was accompanied by reduced thickness of the muscularis externae (Figures 6A,B). Neurofilament-200 (NF200, Nefh) expression was reduced in neurons in *Cntn1*^−/−^ villi compared to wild-type tissues, indicating a defect in axon maturation or innervation (Figure 6C).

**Figure 7:**
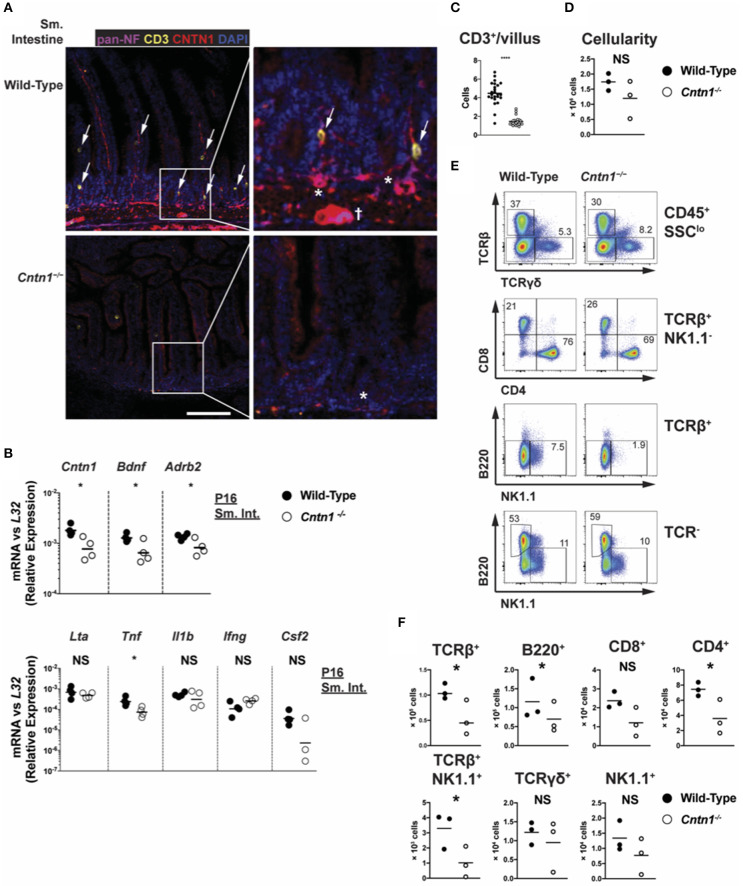
Cntn1 expression within intestinal villi and submucosal/myenteric plexus **(A)**. Representative immunofluorescence images of P11 small intestine from wild-type (top panels) and *Cntn1*^−/−^ (bottom panels) at 4 ×. Tissues were costained for pan-Neurofilament (magenta), CD3 (yellow), Cntn1 (red), and DAPI (blue). Composite images are shown at left with enlarged areas indicated at right. White scale bars represent 50 μm. arrows indicate CD3^+^ cells that are in contact with Cntn1^+^ neurons. * indicates submucosal plexus, dagger indicates myenteric plexus **(B)** Wild-type and *Cntn1*^−/−^ P16 small intestine tissues were harvested for RNA and analyzed for the indicated genes by QRT-PCR. Graphs show transcript abundance relative to *L32*. Individual transcripts were compared by Student's *t*-test **(C)**. The average number of CD3^+^ T cells divided by the average number of villi per visual field was quantified and graphed **(D)**. Graph shows intestine cellularity from wild-type and *Cntn1*^−/−^ animals **(E,F)**. Representative FACS analysis of wild-type (left) and *Cntn1*^−/−^ (right) P12/P13 intestinal lymphocytes. Total live intestinal lymphocytes analyzed for the frequency of αβ T cells (TCRβ^+^) and γδ T cells (TCRγδ^+^), CD4^+^ and CD8^+^ αβ T cells, NK1.1^+^TCRβ^+^ cells, natural killer cells (TCRβ^−^NK1.1^+^), and B cells (B220^+^) **(E)**. The cellularity of each lymphocyte population is graphed in **(F)**. Analysis of data by Student's *t*-test. NS, not significant; *p < 0.05; ****p < 0.0001.

We next examined Contactin-1 distribution within gut tissue. In the small intestine of P11 animals, at which time thymic glucocorticoid signaling is observed, Contactin-1 is expressed in the villus-innervating neurons, as well as in submucosal neurons, including the smaller submucosal plexus (Meissner's plexus) and the larger myenteric plexus (Auerbach's plexus) (Figure 7A) (26). Contactin-1 antibodies costained with a pan-Neurofilament marker in these regions, confirming neuron-specific expression that was absent in *Cntn1*^−/−^ tissues (Figure 7B). Pan-Neurofilament staining was greatly reduced in *Cntn1*^−/−^ tissues, indicating that Contactin-1 is required for axonal maturation/neuronal homeostasis. Reduced smooth muscle thickness in *Cntn1*^−/−^ tissues and Contactin-1 association with submucosal and myenteric plexus neurons indicated a role in regulating neuronal interactions with intestinal smooth muscle. We thus assessed the expression of the neurotrophic factor Brain Derived Neurotrophic Factor (*Bdnf* ) that is expressed in neurons and smooth muscle of the intestine as well as β-2 Adrenergic Receptor (*Adrb2*) that regulates smooth muscle contraction in the intestine (27–29). In P16 *Cntn1*^−/−^ intestines the expression of both *Bdnf* and *Adrb2* were reduced, consistent with decreased neuromuscular crosstalk.

We additionally observed that CD3^+^ T cells within the villi of wild-type animals accumulate in Contactin-1-positive regions, while in *Cntn1*^−/−^ animals the frequency of CD3^+^ T cells per villus was greatly diminished (Figure 7C). In *Cntn1*^−/−^ animals, the small intestine-associated Peyer's Patches showed a trend for reduced cellularity compared to wild-type tissues, and within these lymphoid structures the number of CD4^+^ T cells, TCR-β^+^NK1.1^+^ cells, and B cells was significantly reduced in *Cntn1*-deficient tissues (Figures 7D–F). Additionally, in *Cntn1*^−/−^ intestines *Tnf* expression was significantly lessened, indicating no ongoing inflammation in the gut, consistent with histological and cytometric observations (Figures 1D, 6A, 7B). Together, these data indicate that Contactin-1 is required for optimal maintenance of neuromuscular and immune homeostasis within the intestinal mucosa and loss-of function leads to malnutrition and subsequent organ dysfunction.

## Discussion

In this study, we have set out to characterize how *Cntn1*-deficiency impacts systemic immune homeostasis. *Cntn1*-animals show immune defects including thymic and splenic atrophy and increased expression of inflammatory biomarkers in the spleen. However, ablation of individual cell subsets such as CD4^+^ T cells or macrophages, or of TNF-α did not prevent a wasting phenotype associated with cachexia. In contrast, the changes we observed in blood metabolites and in hematologic cell subsets indicated that early lethality was associated with malnourishment. Additionally, our observation of increased *Gilz* expression in *Cntn1*-null thymuses indicated ongoing glucocorticoid signaling, consistent with hyperactivation of the hypothalamic-pituitary-adrenal (HPA) axis, which has been directly linked with thymocyte apoptosis (19, 22, 30). Reduced NF200/pan-Neurofilament staining in enteric neurons in *Cntn1*^−/−^ tissues that are normally associated with smooth muscle in the small intestine, as well as reduced expression of *Bdnf* and *Adrb2* indicated a defect in neuromuscular crosstalk. Together, these data support the hypothesis that lethality of *Cntn1*-null animals results from malnourishment, that additionally manifests in secondary immune defects. Furthermore, we additionally uncovered a role for Contactin-1 in neuro-immune crosstalk through recruitment of T cells to intestinal villi.

The identified roles of Contactin-1 in the temporal regulation of gut activity and subsequent immune deficiency at approximately 2–3 weeks post birth provide novel aspects of Contactin-1 function. Previous work has identified in *Cntn1*^−/−^ animals defects in axon and dendrite organization that disrupt cerebellar and hypothalamic microorganization (6, 14). While these central defects likely contribute to the ataxic and anorexic phenotype of the *Cntn1-*deficient mice, they cannot explain animal wasting when additional nutrition is provided. The results reported here indicate an additional and novel role for Contactin-1 in mediating the complex crosstalk between neurons, the gut, and the immune system. Thymic involution is a well-characterized physiologic response to systemic stresses including a reduction in food intake (16). Stresses induce activation of the HPA axis in order to release energy stores in tissues throughout the body (18, 30). Glucocorticoids released by the adrenal glands directly mediate thymic atrophy *in vivo* through apoptosis of glucocorticoid receptor-expressing thymocytes (31). It has been suggested that these effects are more pronounced in rodents than in humans (16). Adequate dietary supplementation should correct HPA imbalances and glucocorticoid signaling, and subsequent immune deficiencies in *Cntn1*-null animals. However, a role of Cntn1 in the direct regulation of thymic apoptosis in addition to its role in the enteric nervous system remains to be examined. We have been unsuccessful in rescuing these animals from lethality using manual feeding or parenteral nutrient supplementation, indicating a central role of Contactin-1 in gut function and nutrient uptake.

Contactin-1 was found in the neuromuscular junction in skeletal muscle of mice and humans, although it's function at this cellular location has not been studied further (10). In smooth muscle, innervating neurons can activate individual cells or cell groups through neurotransmitter release from varicosities localized along the axon. Contactin-1 may regulate the development or function of submucosal/myenteric plexus neurons in the intestine, potentially impacting gut contractility and peristaltic function as well as neuronal regulation of nutrient absorption, blood flow and intestinal secretions (32). A recent genome-wide association study identified a number of SNPs within the *CNTN1* gene that were linked with the pathologic *LRRK2* G2019S mutation in a cohort of Parkinson's Disease (PD) patients (33). This report additionally identified a novel PD-associated SNP in the *CNTNAP5* gene that produces an orphan protein proposed to have interactions with Contactin family receptors (1). Interestingly, submucosal and myenteric neurons were found to be atrophied in PD, indicating that these structures and enteric nervous system function are compromised (34). The role of Contactin-1 in the regulation of neuromuscular function in the enteric nervous system, and how defects in this pathway result in disease remain to be fully evaluated. Additionally, *Cntn1*-deficiency may impact neuronal regulation of nutrient absorption by gut epithelium, although we have not directly assessed epithelial cell function. Additionally, we cannot rule out that loss of Contactin-1 in the central nervous system may result in defective food sensing and feeding behavior, resulting in a wasting phenotype.

We observed that Contactin-1 is involved in optimal immune homeostasis within the gut, and perhaps in peripheral lymphoid organs. The development of intestinal lymphocytes is largely complete at birth (35). Our data indicates that while overall cellularity of the intestine is somewhat impacted, the recruitment of CD3^+^ cells to the villi is severely reduced. It is not clear how Contactin-1 functions in this role. Contactin-1 interacts with several ligands that are expressed on T cells including Notch family members and signaling components (36, 37). Alternatively, Contactin-1 signaling may be required to express factors required for T cell recruitment. Notch signaling has been associated with many aspects of immune system function, including development of T cells in the thymus and differentiation of innate and adaptive lymphocytes in the periphery (38). Recently, gut epithelial-expressed Notch receptors were reported to regulate T cell immune function in a model of inflammatory bowel disease (39). It remains unclear, however, whether neuron-expressed Contactin-1 directly activates lymphocyte- or epithelial-expressed Notch receptors. Immune homeostasis within the gut is required for appropriate immune-microbial crosstalk that facilitates development of tolerance to oral antigens, and to prevent autoimmunity (40). It was recently demonstrated that immune responses to gut antigens and specifically the production of antigen-specific IgA antibodies were compromised in fasting animals (41). The development of appropriate IgA antibodies is critical to maintaining microbial homeostasis, prevention of pathogenic infections, and tolerance induction and the prevention of autoimmunity (42). Thus, our data support the idea that Contactin-1 impacts multiple systemic responses that arise from its critical role in peripheral neuron function within the gut.

## Data Availability Statement

The raw data supporting the conclusions of this article will be made available by the authors, without undue reservation, to any qualified researcher.

## Ethics Statement

The animal study was reviewed and approved by Sanford Burnham Prebys Medical Discovery Institute IACUC.

## Author Contributions

MV, CW, BR, and JS contributed to conceptualization and design of the study. MV, DG, KK, WL, JN, SH, and JS performed the experiments, with DG additionally managing animal cohorts and histology, KK and performing confocal microscopy. MV and JS analyzed results and wrote the first draft of the manuscript. DG and KK wrote sections of the manuscript. All authors contributed to manuscript revision, read and approved the submitted version.

## Conflict of Interest

The authors declare that the research was conducted in the absence of any commercial or financial relationships that could be construed as a potential conflict of interest.

## References

[1] Gennarini G Bizzoca A Picocci S Puzzo D Corsi P Furley AJW. The role of Gpi-anchored axonal glycoproteins in neural development and neurological disorders. Mol Cell Neurosci. (2017) 81:49–63. 10.1016/j.mcn.2016.11.006 27871938

[2] Chatterjee M Schild D Teunissen CE. Contactins in the central nervous system: role in health and disease. Neural Regen Res. (2019) 14:206–16. 10.4103/1673-5374.244776 30530999 PMC6301169

[3] Ranscht B. Sequence of contactin, a 130-kD glycoprotein concentrated in areas of interneuronal contact, defines a new member of the immunoglobulin supergene family in the nervous system. J Cell Biol. (1988) 107:1561–73. 10.1083/jcb.107.4.1561 3049624 PMC2115254

[4] Brummendorf T Wolff JM Frank R Rathjen FG. Neural cell recognition molecule F11: homology with fibronectin type III and immunoglobulin type C domains. Neuron. (1989) 2:1351–61. 10.1016/0896-6273(89)90073-1 2627374

[5] Gennarini G Cibelli G Rougon G Mattei MG Goridis C. The mouse neuronal cell surface protein F3: a phosphatidylinositol-anchored member of the immunoglobulin superfamily related to chicken contactin. J Cell Biol. (1989) 109:775–88. 10.1083/jcb.109.2.775 2474555 PMC2115732

[6] Berglund EO Murai KK Fredette B Sekerkova G Marturano B Weber L. Ataxia and abnormal cerebellar microorganization in mice with ablated contactin gene expression. Neuron. (1999) 24:739–50. 10.1016/S0896-6273(00)81126-5 10595523

[7] Boyle ME Berglund EO Murai KK Weber L Peles E Ranscht B. Contactin orchestrates assembly of the septate-like junctions at the paranode in myelinated peripheral nerve. Neuron. (2001) 30:385–97. 10.1016/S0896-6273(01)00296-3 11395001

[8] Colakoglu G Bergstrom-Tyrberg U Berglund EO Ranscht B. Contactin-1 regulates myelination and nodal/paranodal domain organization in the central nervous system. Proc Natl Acad Sci USA. (2014) 111:E394–403. 10.1073/pnas.1313769110 24385581 PMC3903244

[9] Poliak S Peles E. The local differentiation of myelinated axons at nodes of ranvier. Nat Rev Neurosci. (2003) 4:968–80. 10.1038/nrn1253 14682359

[10] Compton AG Albrecht DE Seto JT Cooper ST Ilkovski B Jones KJ. Mutations in contactin-1, a neural adhesion and neuromuscular junction protein, cause a familial form of lethal congenital myopathy. Am J Hum Genet. (2008) 83:714–24. 10.1016/j.ajhg.2008.10.022 19026398 PMC2668069

[11] Vural A Doppler K Meinl E. Autoantibodies against the node of ranvier in seropositive chronic inflammatory demyelinating polyneuropathy: diagnostic, pathogenic, and therapeutic relevance. Front Immunol. (2018) 9:1029. 10.3389/fimmu.2018.01029 29867996 PMC5960694

[12] Chen DH Yu JW Jiang BJ. Contactin 1: A potential therapeutic target and biomarker in gastric cancer. World J Gastroenterol. (2015) 21:9707–16. 10.3748/wjg.v21.i33.9707 26361417 PMC4562954

[13] Zhang R Xie L Liu C Yang H Lin H Zhang Q. Contactin-1: a promising progression biomarker and therapeutic target of carcinoma. Minerva Med. (2017) 108:193–5. 10.23736/S0026-4806.16.04683-8 28222587

[14] Fetissov SO Bergstrom U Johansen JE Hokfelt T Schalling M Ranscht B. Alterations of arcuate nucleus neuropeptidergic development in contactin-deficient mice: comparison with anorexia and food-deprived mice. Eur J Neurosci. (2005) 22:3217–28. 10.1111/j.1460-9568.2005.04513.x 16367788

[15] Rooney I Butrovich K Ware CF. Expression of lymphotoxins and their receptor-Fc fusion proteins by *Baculovirus*. Meth Enzymol. (2000) 322:345–63. 10.1016/S0076-6879(00)22032-6 10914029

[16] Dourov N. Thymic atrophy and immune deficiency in malnutrition. Curr Top Pathol. (1986) 75:127–50. 10.1007/978-3-642-82480-7_4 3514157

[17] Abramson J Anderson G. Thymic epithelial cells. Annu Rev Immunol. (2017) 35:85–118. 10.1146/annurev-immunol-051116-052320 28226225

[18] Coderre L Srivastava AK Chiasson JL. Role of glucocorticoid in the regulation of glycogen metabolism in skeletal muscle. Am J Physiol. (1991) 260:E927–32. 10.1152/ajpendo.1991.260.6.E927 1905485

[19] Gruver AL Sempowski GD. Cytokines, leptin, and stress-induced thymic atrophy. J Leukoc Biol. (2008) 84:915–23. 10.1189/jlb.0108025 18495786 PMC2538595

[20] Howard JK Lord GM Matarese G Vendetti S Ghatei MA Ritter MA. Leptin protects mice from starvation-induced lymphoid atrophy and increases thymic cellularity in ob/ob mice. J Clin Invest. (1999) 104:1051–9. 10.1172/JCI6762 10525043 PMC408574

[21] D'adamio F Zollo O Moraca R Ayroldi E Bruscoli S Bartoli A. A new dexamethasone-induced gene of the leucine zipper family protects T lymphocytes from TCR/CD3-activated cell death. Immunity. (1997) 7:803–12. 10.1016/S1074-7613(00)80398-2 9430225

[22] Delfino DV Agostini M Spinicelli S Vito P Riccardi C. Decrease of Bcl-xL and augmentation of thymocyte apoptosis in GILZ overexpressing transgenic mice. Blood. (2004) 104:4134–41. 10.1182/blood-2004-03-0920 15319285

[23] Ronchetti S Migliorati G Riccardi C. GILZ as a mediator of the anti-inflammatory effects of glucocorticoids. Front Endocrinol. (2015) 6:170. 10.3389/fendo.2015.00170 26617572 PMC4637413

[24] Longhi MP Trumpfheller C Idoyaga J Caskey M Matos I Kluger C. Dendritic cells require a systemic type I interferon response to mature and induce CD4+ Th1 immunity with poly IC as adjuvant. J Exp Med. (2009) 206:1589–602. 10.1084/jem.20090247 19564349 PMC2715098

[25] Koopman FA Stoof SP Straub RH Van Maanen MA Vervoordeldonk MJ Tak PP. Restoring the balance of the autonomic nervous system as an innovative approach to the treatment of rheumatoid arthritis. Mol Med. (2011) 17:937–48. 10.2119/molmed.2011.00065 21607292 PMC3188868

[26] Rao M Gershon MD. The bowel and beyond: the enteric nervous system in neurological disorders. Nat Rev Gastroenterol Hepatol. (2016) 13:517–28. 10.1038/nrgastro.2016.107 27435372 PMC5005185

[27] Lommatzsch M Braun A Mannsfeldt A Botchkarev VA Botchkareva NV Paus R. Abundant production of brain-derived neurotrophic factor by adult visceral epithelia. Implications for paracrine and target-derived neurotrophic functions. Am J Pathol. (1999) 155:1183–93. 10.1016/S0002-9440(10)65221-2 10514401 PMC1867012

[28] Purves D Williams SM Neuroscience. Mass, MI: Sinauer Associates, Sunderland (2001).

[29] Fox EA Biddinger JE. Early postnatal overnutrition: potential roles of gastrointestinal vagal afferents and brain-derived neurotrophic factor. Physiol Behav. (2012) 106:400–12. 10.1016/j.physbeh.2012.04.002 22712064 PMC3517218

[30] Holsboer F Ising M. Stress hormone regulation: biological role and translation into therapy. Annu Rev Psychol. (2010) 61:81–>109, C101–11. 10.1146/annurev.psych.093008.100321 19575614

[31] Brewer JA Kanagawa O Sleckman BP Muglia LJ. Thymocyte apoptosis induced by T cell activation is mediated by glucocorticoids *in vivo*. J Immunol. (2002) 169:1837–43. 10.4049/jimmunol.169.4.1837 12165507

[32] Huh JR Veiga-Fernandes H. Neuroimmune circuits in inter-organ communication. Nat Rev Immunol. (2020) 20:217–28. 10.1038/s41577-019-0247-z 31848462

[33] Vacic V Ozelius LJ Clark LN Bar-Shira A Gana-Weisz M Gurevich T. Genome-wide mapping of IBD segments in an ashkenazi PD cohort identifies associated haplotypes. Hum Mol Genet. (2014) 23:4693–702. 10.1093/hmg/ddu158 24842889 PMC4119402

[34] Ohlsson B Englund E. Atrophic myenteric and submucosal neurons are observed in Parkinson's disease. Parkinsons Dis. (2019) 2019:7935820. 10.1155/2019/7935820 31321021 PMC6607708

[35] Cheroutre H Lambolez F Mucida D. The light and dark sides of intestinal intraepithelial lymphocytes. Nat Rev Immunol. (2011) 11:445–56. 10.1038/nri3007 21681197 PMC3140792

[36] Hu QD Ang BT Karsak M Hu WP Cui XY Duka T. F3/contactin acts as a functional ligand for Notch during oligodendrocyte maturation. Cell. (2003) 115:163–75. 10.1016/S0092-8674(03)00810-9 14567914

[37] Hur JY Teranishi Y Kihara T Yamamoto NG Inoue M Hosia W. Identification of novel γ-secretase-associated proteins in detergent-resistant membranes from brain. J Biol Chem. (2012) 287:11991–2005. 10.1074/jbc.M111.246074 22315232 PMC3320946

[38] Radtke F Macdonald HR Tacchini-Cottier F. Regulation of innate and adaptive immunity by notch. Nat Rev Immunol. (2013) 13:427–37. 10.1038/nri3445 23665520

[39] Mathern DR Laitman LE Hovhannisyan Z Dunkin D Farsio S Malik TJ. Mouse and human Notch-1 regulate mucosal immune responses. Mucosal Immunol. (2014) 7:995–1005. 10.1038/mi.2013.118 24424521

[40] Veiga-Fernandes H Mucida D. Neuro-immune interactions at barrier surfaces. Cell. (2016) 165:801–11. 10.1016/j.cell.2016.04.041 27153494 PMC4871617

[41] Nagai M Noguchi R Takahashi D Morikawa T Koshida K Komiyama S. Fasting-refeeding impacts immune cell dynamics and mucosal immune responses. Cell. (2019) 178:1072–87 e1014. 10.1016/j.cell.2019.07.047 31442401

[42] Honda K Littman DR. The microbiota in adaptive immune homeostasis and disease. Nature. (2016) 535:75–84. 10.1038/nature18848 27383982

